# Electrical Impedance Tomography Can Be Used to Quantify Lung Hyperinflation during HFOV: The Pilot Study in Pigs

**DOI:** 10.3390/diagnostics12092081

**Published:** 2022-08-28

**Authors:** Vaclav Ort, Karel Roubik

**Affiliations:** Department of Biomedical Technology, Faculty of Biomedical Engineering, Czech Technical University in Prague, nam. Sitna 3105, 272 01 Kladno, Czech Republic

**Keywords:** dynamic hyperinflation, dynamic hypoinflation, electrical impedance tomography, high-frequency oscillatory ventilation

## Abstract

Dynamic hyperinflation is reported as a potential risk during high-frequency oscillatory ventilation (HFOV), and its existence has been documented both by physical models and by CT. The aim of this study is to determine the suitability of electrical impendence tomography (EIT) for the measurement of dynamic lung hyperinflation and hypoinflation during HFOV. Eleven healthy pigs were anaesthetized and ventilated using HFOV. The difference between the airway pressure at the airway opening and alveolar space was measured by EIT and esophageal balloons at three mean airway pressures (12, 18 and 24 cm H_2_O) and two inspiratory to expiratory time ratios (1:1, 1:2). The I:E ratio was the primary parameter associated with differences between airway and alveolar pressures. All animals showed hyperinflation at a 1:1 ratio (median 1.9 cm H_2_O) and hypoinflation at a 1:2 (median –4.0 cm H_2_O) as measured by EIT. EIT measurements had a linear correlation to esophageal balloon measurements (r^2^ = –0.915, *p* = 0.0085). EIT measurements were slightly higher than that of the esophageal balloon transducer with the mean difference of 0.57 cm H_2_O. Presence of a hyperinflation or hypoinflation was also confirmed independently by chest X-ray. We found that dynamic hyperinflation developed during HFOV may be detected and characterized noninvasively by EIT.

## 1. Introduction

Mechanical ventilation is an effective therapy in patients with severe respiratory impairment or respiratory failure, but it may cause severe damage to the respiratory system (RS) [1,2]. High-frequency oscillatory ventilation (HFOV) is an unconventional ventilatory technique that may minimize the adverse effects of mechanical ventilation by using very small tidal volumes and pressure amplitudes preventing volumotrauma and barotrauma of the lungs [3,4,5]. The initial ventilator setting and optimization of HFOV are still empirical and require experienced personnel, which represents a disadvantage of HFOV. Another essential problem associated with HFOV use in clinical setup is the inaccessibility of delivered tidal volume (VT) and alveolar pressure (Palv) values. HFOV is usually set to a desired mean airway pressure (mPaw), which can be measured easily [6] but which can be different from the mean alveolar pressure (mPalv) [6,7,8].

Several studies have documented differences between mPalv and mPaw. This is referred to as the dynamic hyperinflation (DH) [6]. The DH develops due to an insufficient time for the expiration, truncated by the subsequent inspiration [9,10]. The magnitude of DH is referred to as auto-PEEP in the case of conventional ventilation. However, due to the unclear definition of PEEP in HFOV, we do not use this term and remain with the term “magnitude of DH”. The volume of the lungs is therefore increased by a certain volume of gas. This situation is referred to as “air-trapping” [11]. Air trapping at the end of expiration or end of inspiration can cause the barotrauma and a cardiovascular compromise [12].

Several studies documented that mPalv during HFOV may be higher [6,10,13], equal to [7,14,15], or even lower [7,15] than mPaw. Since the last condition is the opposite of DH, we refer to this state of the RS as the dynamic hypoinflation.

The DH can be measured by the occlusion maneuver using an esophageal balloon [16] and, in laboratory animals, by the alveolar capsule technique [8]. Air-trapping during HFOV can be also assessed by CT [7]. None of the above methods of DH measurement is suitable for a routine application in clinical practice due to their invasiveness, use of ionizing radiation or interference with a ventilatory support.

In their study, Adler et al. [17] showed that DH can be measured by electrical impedance tomography (EIT) during conventional ventilation. They also found that the measurement of volume of air trapped in the lungs using EIT is more precise than techniques based on the occlusion maneuver or the esophageal pressure measurement.

EIT is a noninvasive, easy-to-use and radiation-free technique suitable for bedside continuous monitoring of the lungs [18]. EIT is an imaging modality that provides information about regional lung ventilation. It is based on the application of small alternating currents using skin electrodes attached to the patient’s chest and the consequent measurement of resulting voltages. EIT is also suitable for dynamic measuring of alveolar overdistension [19]. Another potential benefit of EIT is a possibility of alveolar pressure (Palv) measurement and DH assessment. The possibility of Palv measurement by EIT may represent a simple method for lung overdistention monitoring [17] and determining the optimal HFOV setting, the importance of which has been identified recently [20]. 

The aim of the study is to investigate whether EIT represents a suitable method for dynamic hyperinflation measurement during HFOV and to design a method of assessment of a dynamic hyperinflation or a dynamic hypoinflation in a porcine model.

## 2. Materials and Methods

The prospective interventional animal study was approved by the Institutional Review Board of the First Faculty of Medicine, Charles University in Prague (FFM CU), on 27 March 2013, in accordance with the Act No. 246/1992 Coll., on the protection of animals against cruelty. The measurements were performed in an accredited animal laboratory of the FFM CU. All methods were performed in accordance with the relevant guidelines and regulations. The authors complied with the ARRIVE (Animal Research: Reporting of In Vivo Experiments) guidelines. 

Eleven healthy female pigs (*Sus scrofa domestica*) weighing 42 kg (SD 3 kg) were included in the study. The anesthesia and animal care followed the protocol approved for the animal laboratory and was used in other studies in pigs [21,22].

The animals were premedicated with midazolam (0.3 mg·kg^−1^ IM). Anesthesia was initiated with ketamine hydrochloride (20 mg·kg^−1^ IM) followed by boli of morphine (0.1 mg·kg^−1^ IV) and propofol (2 mg·kg^−1^ IV). A cuffed endotracheal tube (I.D. 7.5 mm) was used for intubation. Anesthesia was maintained with propofol (8 to 10 mg·kg^−1^·h^−1^ IV) in combination with midazolam (0.2–0.4 mg·kg^−1^·h^−1^ IV) and morphine (0.1 mg·kg^−1^·h^−1^ IV). Coagulation was prevented by heparin (40 U·kg^−1^·h^−1^ IV). To suppress spontaneous breathing, myorelaxant pipecuronium bromide (4 mg bolus every 45 min) was administered during mechanical lung ventilation (Hamilton G5, Hamilton Medical AG, Bonaduz, Switzerland). The esophageal balloon (The adult esophageal balloon catheter kit, Hamilton Medical AG, Bonaduz, Switzerland) was installed. Infusion of crystalloids was continually administered to reach and maintain central venous pressure of 4 to 5 mm Hg (typically 3–6 mL·kg^−1^·h^−1^ IV). Vital signs of the animal were monitored using Carescape B650 (GE Healthcare, Chicago, IL, USA) monitor. Arterial blood gases, i.e., arterial partial pressure of oxygen, carbon dioxide and pH, were continuously measured by CDI 500 (Terumo, Tokyo, Japan). The arterio–venous extracorporeal circuit for CDI 500 monitor was established between the femoral artery and the femoral vein using a mechanical blood pump (peristaltic roller pump with a blood flow set to 400 mL·min^−1^).

**Figure 1:**
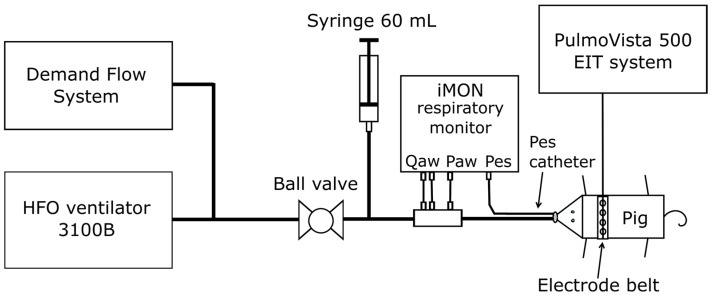
Scheme of the experimental assembly.

The EIT electrode belt was placed on the chest of the animal at the level of the 5th–6th intercostal space confirmed by chest X-ray. The electrode belt was connected to the PulmoVista 500 EIT system (Dräger Medical, Lübeck, Germany), and the EIT measurement with data recording was initiated. In this study, the global impedance calculated from the recorded data using Draeger EIT Analysis Tool 6.1 (Dräger Medical, Lübeck, Germany) was used as the EIT output signal. The animal was switched to HFOV ventilator 3100B (CareFusion, Yorba Linda, CA, USA), and a custom-made monitoring device iMON [23] followed by a ball valve was introduced between the patient circuit and the ETT as depicted in Figure 1. The iMON monitor recorded airway pressure (Paw), mean airway pressure (mPaw) measured at the proximal end of the ETT, esophageal pressure (Pes), airflow and tidal volume during HFOV. In order to keep mPaw constant during the entire experiment, a demand flow system (DFS) [24] was connected to the patient circuit.

The initial setting of HFOV ventilator was as follows: mPaw 18 cm H_2_O, bias flow 25 L·min^−1^, oscillatory frequency 5 Hz and inspiratory to expiratory time ratio (I:E) 1:1. Amplitude of oscillations (ΔP) was set to maintain normocapnia (35–45 mm Hg). Fraction of oxygen (FiO_2_) was slightly elevated (between 0.21–0.3) to reach normoxemia (80–90 mm Hg).

**Figure 2:**
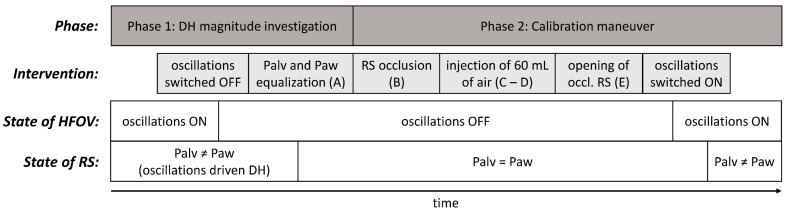
Phases of the experiment and the interventions performed.

Measurements were performed at mPaw levels of 12, 18 and, if tolerated by an animal, 24 cm H_2_O with two different I:E ratios of 1:1 and 1:2. These two I:E ratios represent the margin values of the interval that can be set on the 3100B ventilator. The whole experiment for each combination of mPaw and I:E had two phases, as depicted in Figure 2.

**Figure 3:**
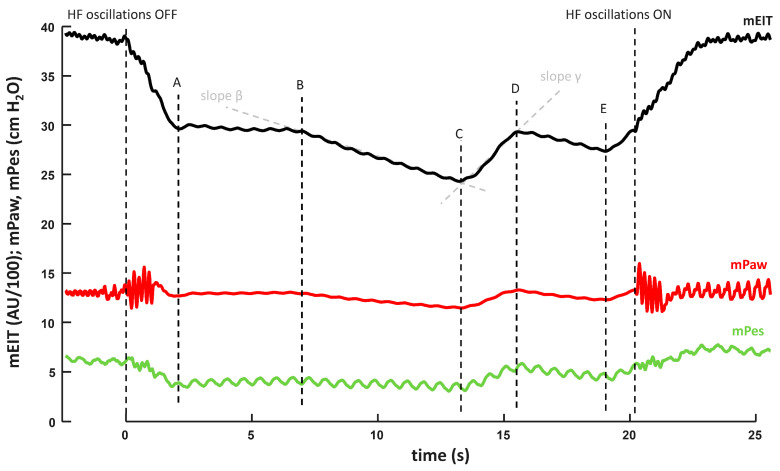
The signals evaluated during calibration and DH measurement. (A–B) Equilibrium between Paw (maintained by the Demand Flow System) and Palv after oscillations were turned off; (B) occlusion of the RS using the two-way valve; (B–C) pressure drop caused by an air leak after the airway occlusion; (C–D) injection of 60 mL of air; (D–E) a pressure drop caused by the air leak after the airway occlusion; (E) reopening of the occluded airways and pressure returning to a level maintained by the demand flow system.

First, the oscillations of the HFOV ventilator were stopped, while mPaw was maintained constant using the DFS. The cessation of the oscillations led to the equalization of the mPalv to mPaw, resulting in changes in Pes and EIT signals; see Figure 3. Since mPaw was exactly the same with and without the oscillation, the observed change in Pes and EIT should correspond to the magnitude of DH.

Second, a calibration maneuver was conducted in order to facilitate the recalculation of changes in EIT and Pes to the actual changes in Palv, i.e., to pressure changes in the alveolar space. In this calibration maneuver, 60 mL of air was injected into the occluded RS, which corresponds to the segment CD in Figure 3. The air injection caused an increase in lung volume and an increase in the equalized pressure (Palv = Paw) in the occluded RS, which was manifested by an increase in both Paw and EIT signal. Since this change was caused by the same maneuver in both the signals, the magnitude of these changes for a two-point pressure calibration of the EIT signal can be used. Subsequently, it is possible to recalculate the EIT signal changes to the pressure changes in the occluded RS resulting from the cessation of DH due to the switching off of the oscillations. The trends of mPaw and EIT signal during interventions are depicted in Figure 3. The same two-point method was used for the Pes calibration.

Next, after the air leak compensation (calculated from the slopes β and γ, see Figure 3), the trapped volume of air during DH was calculated from EIT. In order to verify the measurement method, the correctness of the trapped volume calculation was verified by an injection (in case of the recorded dynamic hypoinflation) or removal (in case of the dynamic hyperinflation) of previously calculated trapped air volume into/from the occluded RS. The measurement was supposed to be correct if the EIT signal after this testing maneuver and EIT signal before switching the oscillations off were equal and, moreover, if the Paw measured by the iMON device after this maneuver reached the same level as the mPalv calculated from the EIT during ventilation.

As an independent method for confirmation of dynamic hyperinflation or hypoinflation existence, a chest X-ray was used during the measurements. As tidal volumes and pressure amplitudes in the alveolar space during HFOV are very small in comparison with conventional mechanical ventilation [25,26,27,28], the diaphragm do not move significantly during HFOV breathing cycles, and therefore there is no need for a synchronization of the X-ray imaging with a certain phase of the breathing cycle.

The primary measures of the study were differences between mPaw measured at the airway opening and mPalv assessed by both EIT and the esophageal balloon catheter. The analyses and comparisons were conducted in MATLAB (MathWorks, Natick, MA, USA) and Statistica 7.1 (TIBCO Software Inc., Palo Alto, CA, USA) software. Statistical significance of the DH magnitude differences at various mPaw levels were tested using the repeated measures ANOVA after the Shapiro-Wilk data normality test (Statistica 7.1). *p* < 0.05 was considered as statistically significant. Finally, the magnitude of DH calculated from changes in EIT and Pes were evaluated using the Bland-Altman analysis.

## 3. Results

All 11 animals completed the entire protocol and were included in the analysis. From these 11 animals, only 5 animals tolerated the highest preset mPaw level of 24 cm H_2_O. 

**Figure 4:**
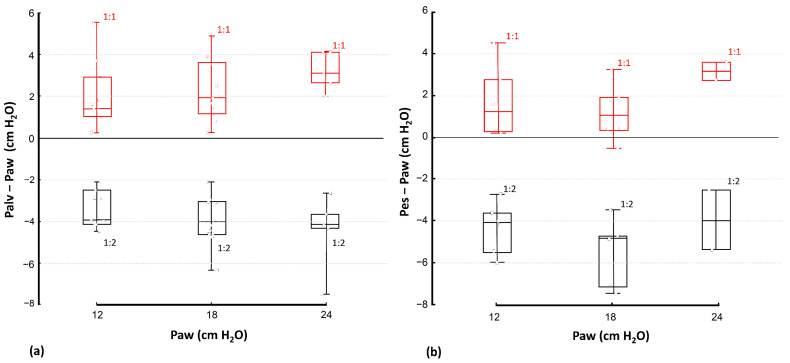
Comparison of DH evaluated by two different methods: (**a**) DH_EIT_ determined as Palv (measured using EIT) minus Paw, and (**b**) DH_Pes_ determined as Pes (measured using esophageal balloon) minus Paw.

Development of dynamic hyperinflation was strongly dependent on the inspiratory to expiratory time ratio, as documented in Figure 4. In all the animals, dynamic hyperinflation developed at the inspiratory to expiratory time ratio of 1:1 at all preset mPaw levels, whereas dynamic hypoinflation occurred in all animals at the inspiratory to expiratory time ratio of 1:2 at all preset mPaw levels. The range of measured dynamic hyperinflation was 0.3–5.5 cm H_2_O (median 1.9 cm H_2_O) for I:E = 1:1, and the range was from –7.5 to –2.1 cm H_2_O (median –4.0 cm H_2_O) for I:E = 1:2. There was no statistically significant dependence of DH magnitude on the preset mPaw levels.

**Figure 5:**
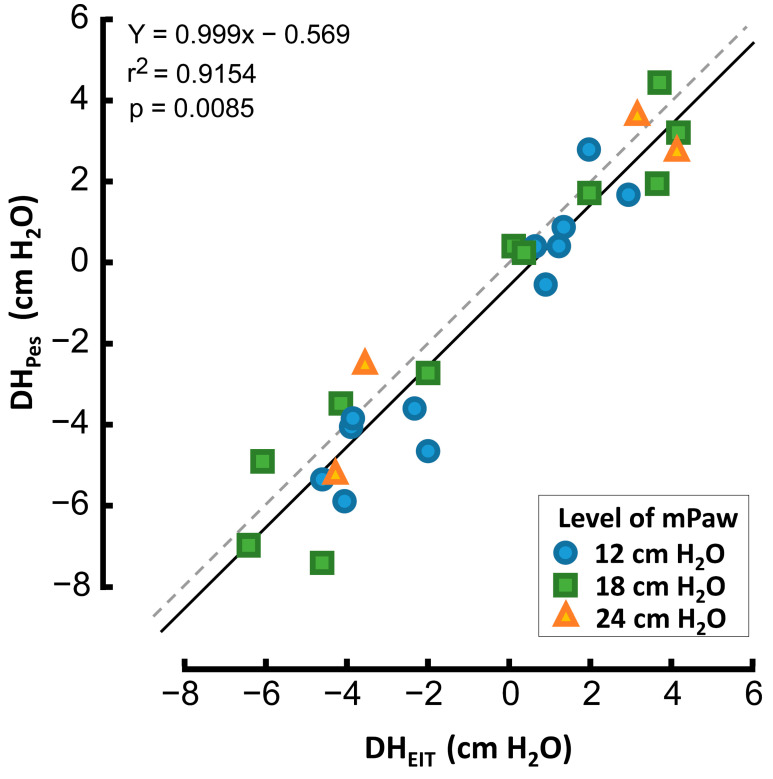
Correlation between DH_EIT_ (determined using EIT) and DH_Pes_ (determined using esophageal balloon). Dotted line represents the 45-degree line.

**Figure 6:**
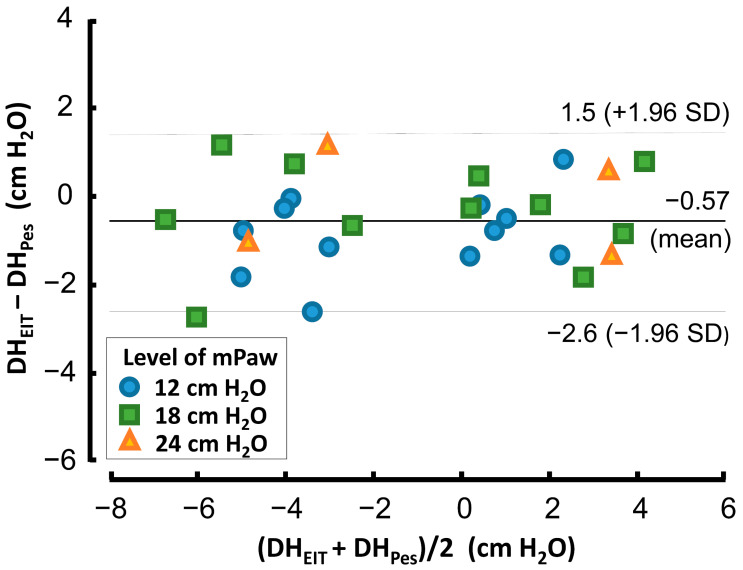
Bland-Altman analysis of two methods used for DH measurement: DH_EIT_ measured by EIT and DH_Pes_ measured by esophageal balloon. The dashed lines show the 95% limits of agreement.

Magnitudes of DH measured by EIT correlated well with the values measured by esophageal balloon. The linear relationship between these two variables was expressed as a linear equation Palv = 0.999·Pes − 0.57 with the coefficient of determination r^2^ = 0.915 (Figure 5). The Bland-Altman plot, presented in Figure 6, shows the 95% limits of agreement between both methods. The differences between results of these two methods lay between –2.6 cm H_2_O and 1.5 cm H_2_O. There was a systematic difference between these two methods, where DH measured by EIT (DH_EIT_) yielded values lower by 0.57 cm H_2_O than DH measured by esophageal balloons (DH_PES_).

**Figure 7:**
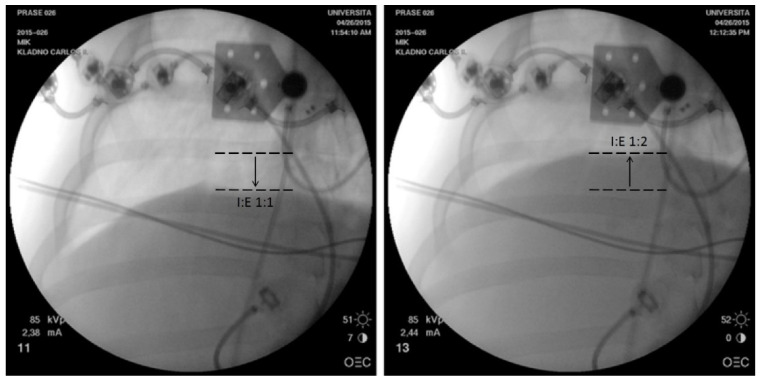
Diaphragm position at I:E time ratios of 1:1 (**left**) and 1:2 (**right**) in a pig at Paw of 18 cm H_2_O. The different positions of the diaphragm at I:E 1:1 and 1:2 are indicated by dashed lines in the figure. No change was made to the magnification or distance above the animal at which the images were taken, and all images were taken in an unchanged postero–anterior position.

Presence of dynamic hyperinflation or dynamic hypoinflation was confirmed independently by a chest X-ray. Change of the I:E time ratio from 1:1 to 1:2 at all Paw levels caused a significant diaphragm elevation, as shown in Figure 7, for one animal at Paw of 18 cm H_2_O. The similar diaphragm shifts were recorded in all the animals included in the study. The observed change in diaphragm position was significantly greater than the diaphragm movements caused by small HFOV tidal volumes. The diaphragm basically does not change its position during HFOV inspiration and expiration. For this reason, the observed change of diaphragm position presented in Figure 7 is related to DH.

## 4. Discussion

The main finding of this study is that dynamic hyperinflation and dynamic hypoinflation, which develops during HFOV, can be confirmed and measured by EIT. Development and magnitude of dynamic hyperinflation and dynamic hypoinflation is strongly dependent on and determined by the inspiratory to expiratory time ratio I:E.

Dynamic hyperinflation (mPalv > mPaw) occurred at I:E = 1:1, and dynamic hypoinflation (mPalv < mPaw) occurred at I:E = 1:2. The magnitude of both dynamic hyperinflation (from 0.3 to 5.5 cm H_2_O, median 1.9 cm H_2_O) and dynamic hypoinflation (from –7.5 to –2.1 cm H_2_O, median –4.0 cm H_2_O) detected in our study were reasonable and may be of clinical importance. There was no significant effect of preset mPaw on the magnitude of dynamic hyperinflation or hypoinflation.

The results of the current study correspond with the previously published studies. In these studies, the development of dynamic hyperinflation [6,10,13], dynamic hypoinflation (i.e., mPaw > mPalv) [7,15] or equilibrium between mPaw and mPalv [7,14,15] were documented depending on the conditions of measurement. Furthermore, the current study described the ventilator setting likely causing these reported differences. We found that the I:E seems to be the most influential parameter as I:E 1:1 always caused dynamic hyperinflation, and I:E = 1:2 always caused dynamic hypoinflation. Our results imply that there should be an I:E ratio between 1:1 and 1:2 at which DH does not develop. Determination of such I:E ratio could not be conducted in real-time easily, because the EIT system used in this study did not allow direct access to the measured data during the experiment, and the offline data analysis was necessary. However, finding the approximate I:E at which DH does not occur can be achieved by switching off the oscillations on the HFOV ventilator while maintaining a constant mPaw. When the oscillations are switched off, any DH cease, which is reflected by a change in the EIT signal on the EIT monitor (as shown in Figure 3). If there is no change in the EIT signal after the oscillations are switched off at a certain I:E, DH does not occur at that I:E, or its magnitude is negligible. Nevertheless, to ascertain the accurate DH value for each I:E, data would need to be obtained from the EIT instrument online for a real-time analysis. Another parameter that has been shown to affect the magnitude of dynamic hyperinflation is the amplitude of oscillations (ΔP) [15]. However, ΔP in this study was set to achieve normocapnia, which is more clinically relevant than manipulation with ΔP outside the normocapnic range. Therefore, the effect of ΔP on the magnitude of dynamic hyperinflation was not examined in this study.

The results presented in our study are derived from values of alveolar pressure measured by EIT. The changes in mPalv were calculated from changes of the chest impedance. To facilitate the calculation, a calibration maneuver was conducted. The calibration maneuver consisted of injection of 60 mL of air into the occluded RS (in order to equilibrate Palv and Paw), while Paw and EIT were measured simultaneously, and data for two-point calibration of EIT signal were acquired. Such small volumes of the injected air were used intentionally so that they induced small volume changes in the RS and the RS could be thus considered linear. As a result, the possible errors in calculation of the magnitude of DH measured by EIT were minimized. A two-point calibration of EIT signal for Palv was used. The corresponding reference values of EIT and Palv were measured with the occluded RS and without high-frequency oscillations. The two-point calibration requires a linear relationship between EIT data and mPalv. In order to confirm the linearity in the range of parameters used in our study and to verify the method itself, we conducted several tests.

First, the linearity of chest impedance dependence upon the lung volume was tested using injections of 30, 60, 100 and 200 mL of air into the occluded RS of the animal with simultaneous EIT recording. The impedance increased linearly with the increasing injected volume.

Correctness of the Palv calculation from EIT signal (described in the method section) was also confirmed by esophageal pressure measurement. The results acquired using both the EIT and Pes methods were consistent, as documented in Figure 5 and Figure 6. 

**Figure 8:**
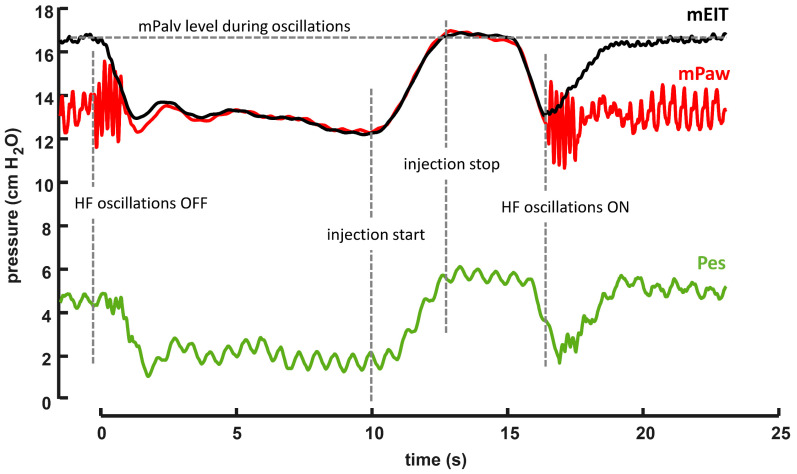
Verification of the calibration maneuver by injection of the calculated air-trapped volume. Calculated air-trapped volume is based on the previous calibration maneuver. The figure shows that after the injection of the calculated volume into the occluded RS with the oscillations stopped, the EIT signal reached the same level as while the oscillations were on. A similar response can be observed on the esophageal pressure signal.

Moreover, after air leak compensation (calculated from slopes β a γ, see Figure 3), the trapped volume of air during DH was calculated from EIT. Correctness of the trapped volume calculation was verified by injection (in case of recorded dynamic hypoinflation) or removal (in case of dynamic hyperinflation) of the previously calculated trapped air volume into/from the occluded RS. The measurement was assumed to be correct if the EIT signal after this testing maneuver was equal to the EIT signal before switching off the oscillations and, moreover, if the Paw measured by the iMON device after this maneuver reached the same level as the mPalv calculated from the EIT during ventilation; see Figure 8. We consider this to be a proof of the functionality of the mPalv measurement method using EIT and the calibration maneuver.

According to the Bland-Altman analysis, DH_Pes_ calculated using Pes was higher by 0.57 cm H_2_O in average, in comparison with alveolar pressure DH_EIT_ calculated using EIT data. We speculate that the observed small difference might be caused as a result of different gas distribution in the lungs during static conditions (i.e., during the calibration without oscillations) and during dynamic conditions (i.e., during HFOV).

Furthermore, esophageal pressure measurement is prone to frequent errors originating from Pes catheter position, body position, calibration, verification, etc. Adler et al. conducted a study on conventional ventilation in dogs [17] in which they found that the average measurement error of lung volume measured by Pes is double than that of measured by EIT. For those reasons, we speculate that the assessment of Palv measured by EIT may be more reliable than using Pes.

Since the difference between values of mPaw and mPalv depends on I:E ratio, the development of dynamic hyperinflation was possible to check visually using chest X-ray images recorded at the same mPaw pressure but with different I:E rations.

This study has several limitations: First, a group of healthy pigs was used for the experiment. The healthy pigs were selected as they represent an animal model stable in time with a small interindividual variability of the lung parameters among the subjects. The aim of the study was to validate the method for DH measurement using EIT; thus, we did not tend to investigate the effect of lung pathology (i.e., ARDS) and other ventilator parameters such as frequency of oscillations, ΔP, etc. Second, the esophageal balloons were introduced into the animals under general anesthesia and muscular relaxation; therefore, the proper position of esophageal balloon could not be verified using the recommended occlusion maneuver. Finally, DFS was used in the study. We use DFS regularly during our animal experiments, and we confirmed that DFS assures a stabile mPaw when switching the oscillations off. DFS has not been proved for clinical use yet; therefore, we are searching for another way to achieve mPaw stabilization during the calibration maneuver. 

The study has direct clinical implication. The study proved that EIT is a suitable method for DH measurement through Palv calculation using EIT data. The main advantages of this method are that it does not use ionizing radiation, does not require interventions as introduction of esophageal balloon, etc. EIT can be used bedside and can be used for long-term lung monitoring. 

Analysis [20] of large multicenter studies OSCAR [29] and OSCILLATE [30] recommends monitoring of Palv, lung recruitment and possibly regional distribution of ventilation both in clinical practice and in future HFOV research studies. According to the study by Adler et al. [17] and the results of the current study, all these recommendations can be met when EIT is used for the lung volume monitoring after the appropriate calibration. In the current study we used only global impedance of the chest for DH characterization. Nevertheless, the described methodology can be easily used to measure regional distribution of DH, as well.

## 5. Conclusions

Dynamic hyperinflation developing during HFOV may be detected and characterized noninvasively by electrical impedance tomography. The pressure difference can be either positive or negative, i.e., dynamic hyperinflation or dynamic hypoinflation may develop depending on inspiratory to expiratory time ratio.

## Data Availability

The data presented in this study are openly available in the repository at https://ventilation.fbmi.cvut.cz/data/ (accessed on 25 August 2022).
